# Symbiont demand guides resource supply: leaf-cutting ants preferentially deliver their harvested fragments to undernourished fungus gardens

**DOI:** 10.1007/s00114-022-01797-7

**Published:** 2022-04-25

**Authors:** Daniela Römer, Gonzalo Pacheco Aguilar, Annika Meyer, Flavio Roces

**Affiliations:** Department of Behavioral Physiology and Sociobiology, Biocenter, University of Würzburg, Am Hubland, 97074 Würzburg, Germany

**Keywords:** Insect-fungus symbiosis, Nutrition, Pheromone trail, Local cues, Decision-making, Decentralized control

## Abstract

Leaf-cutting ants are highly successful herbivores in the Neotropics. They forage large amounts of fresh plant material to nourish a symbiotic fungus that sustains the colony. It is unknown how workers organize the intra-nest distribution of resources, and whether they respond to increasing demands in some fungus gardens by adjusting the amount of delivered resources accordingly. In laboratory experiments, we analyzed the spatial distribution of collected leaf fragments among nest chambers in *Acromyrmex ambiguus* leaf-cutting ants, and how it changed when one of the fungus gardens experienced undernourishment. Plant fragments were evenly distributed among nest chambers when the fungal symbiont was well nourished. That pattern changed when one of the fungus gardens was undernourished and had a higher leaf demand, resulting in more leaf discs delivered to the undernourished fungus garden over at least 2 days after deprivation. Some ants bypassed nourished gardens to directly deliver their resource to the chamber with higher nutritional demand. We hypothesize that cues arising from that chamber might be used for orientation and/or that informed individuals, presumably stemming from the undernourished chamber, may preferentially orient to them.

## Introduction

The success of modern human societies largely depends on a quick and reliable distribution of resources, usually guided by centralized control. Insect societies are often compared to human ones based on their complex organization. However, their social organization is not governed by a central control, but rather emerges from individual responses to local cues. Social insects are central-place foragers, as they collect distant food resources and transport them back to the colony where they are distributed among nestmates. The distribution of fluid food is generally achieved through trophallaxis, the transfer from one individual to another (reviewed by Meurville and LeBoeuf 2021). Resource distribution among nestmates is fast: for example, food was distributed among all 200 members of a *Formica fusca* ant colony within 30 min (Buffin et al. 2009). Food supply depends on specific colony demands (Cassill and Tschinkel 1999). After starvation, foragers collect more food and distribute it among more nestmates (Howard and Tschinkel 1980), or supply liquid food at a faster speed (Mailleux et al. 2011).

Leaf-cutting ants (LCA) are the dominant herbivores of the Neotropics and are able to sustain huge colonies through a unique system: the farming of a symbiotic fungus in underground nest chambers (Moreira et al. 2004). The fungus, food source of the colony brood, grows on the foraged plant material, while the adult workers mostly sustain themselves by ingested plant sap (Bass and Cherrett 1995). When bringing in hundreds of leaf fragments to supply their symbiotic partner, LCA manage to evenly distribute them among all their fungus chambers (Pretto and Forti 2000). To organize resource distribution, LCA should be able to react to changing demands of their symbiotic partner, which might occur due to brood presence or to fungus decline caused by unsuitable environmental conditions or harmful effects from plants previously harvested. In this work, we investigated how LCA distribute the harvested fragments within the nest and explored the flexibility of intra-nest leaf distribution under increased demands of single fungus gardens.

## Methods

The experiments were performed at the University of Würzburg, Germany, with one queenright mature colony of *Acromyrmex ambiguus* (collected 2008, Uruguay), reared in a climate chamber (50% humidity, 25 °C, and 12:12 h LD cycle). The nest consisted of 8 fungus chambers (19 × 19 × 9 cm), 2 waste boxes, and a feeding arena. Each chamber was completely filled with a fungus garden and contained approx. 10^3^ workers and brood. In which chamber the queen resided was unknown. Three chambers were connected to a set-up for the trials, which were done with 8 fungus chambers arranged in 12 different configurations to randomize sampling during data collection. Those chambers that were used more than once remained connected to the mother colony in between the trials for at least 7 days. Only chambers that were completely filled with a fungus garden the day prior to a trial were connected to the setup, as described below.

**Fig. 1:**
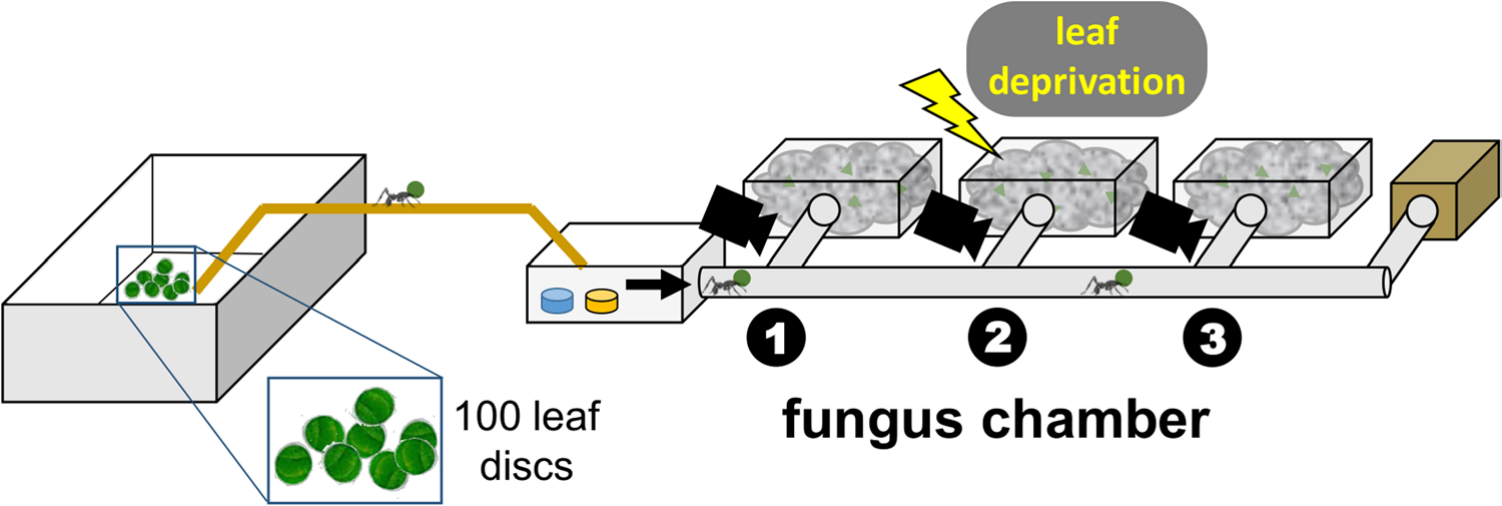
Setup with foraging arena (left) connected by a bridge to a subcolony of 3 fungus chambers and a final waste box (brown). Diluted honey (yellow dish) and water (blue dish) were offered in the small box before the nest entrance (black arrow). The yellow arrow marks the chamber that experienced leaf deprivation in the ‘undernourished’ experiment

The experimental setup consisted of a subcolony composed of 3 fungus chambers, disconnected from the mother colony, and a waste box, connected serially at the sides of a main tunnel via short, lateral tunnels (PVC tube, diameter 2.5 cm; Fig. 1). The nest design resembles the natural arrangement of the multi-chambered, small shallow nests of this species (Bonetto 1959), as well as the serial chamber arrangement known in several other *Acromyrmex* and also all *Atta* LCA species (Bollazzi et al. 2021), in which workers do not need to enter and pass through a fungus chamber in order to reach the subsequent one. The main tunnel connected to the foraging arena (45 × 35 × 9 cm) via an open box (19 × 19 × 9 cm) and a wooden bridge (width 4 cm, length 100 cm). In the open box, water and diluted honey solution were offered ad libitum. Three CCTV video cameras were placed above each of the side tunnels leading to the fungus chambers to later count outgoing and ingoing workers carrying leaf discs. To build up a steady traffic flow, workers were allowed to cut and carry leaf fragments into the nest starting 1 h before each daily trial began.

The general procedure was as follows: a daily trial consisted of three assays, performed at 10:00, 12:00, and 14:00 h, to account for potential daily changes in resource distribution patterns. At the beginning of each assay, 100 leaf discs (6 mm diameter) were placed in the foraging arena, 25 discs every 5 min. An assay lasted 45 min and the number of leaf discs transported into each chamber was counted from the video recordings. As workers relocated some discs between chambers, it was unavoidable to count some discs more than once, as they were not individually marked. Preliminary trials using individually marked leaf discs indicated that no more than 10% of discs were relocated, which could have led to overestimated counts of the same magnitude in our experiments. In addition to the counts, we also scored which fungus garden received the first delivery of the offered leaf discs in each assay.

Two different experiments were performed. In the ‘nourished’ experiment (*n* = 12), we tested whether a similar leaf demand would lead to a uniform supply to all fungus gardens. All three fungus gardens were well nourished as the subcolony received leaves (*Rubus fruticosus*) *ad libitum* prior to each trial. In the ‘undernourished’ experiment (*n* = 13), we tested whether LCA would react to an increased resource demand of a single fungus garden and whether this reaction would last for more than 1 day. Therefore, the ‘undernourished’ experiment consisted of two subsequent daily trials. On the day the first trial started, the original, well-nourished fungus garden located at the middle of the nest was replaced by an undernourished one. The undernourished fungus garden originated from the same mother colony and was previously prepared as follows. Two days prior to the trial, the fungus garden to become undernourished was detached from the well-nourished mother colony and kept isolated, yet connected to a small box where workers were supplied only with diluted honey *ad libitum* and water, and could deposit waste. The isolated fungus garden was not supplied with leaves for the next 48 h, during which it slightly but noticeably decreased in volume, and no longer showed the typical layer of greyish-green, well-supplied growing fungus on top. Thereafter, the undernourished fungus garden was connected to the subcolony 1 h prior to the start of the first daily trial, to allow workers to explore the whole setup. Counts were performed as described above on that and on the subsequent day. Importantly, the subcolony was supplied with some additional fresh leaves after the first daily trial for overnight foraging, to investigate whether the incorporation of leaf fragments collected during both the first trial and the night led to a change in the pattern of resource distribution the following day.

Data were first tested for normality (Shapiro–Wilk test) and for equal variance; thereafter, 2-way repeated-measures ANOVA was performed to analyze distribution patterns and a possible dependence on time of day. Afterwards, as either normality or equal variance was not met for the pooled data of the ‘undernourished’ experiment, analyses using a Kruskal–Wallis test and a Tukey post hoc test were performed. The first delivery of offered discs was analyzed using Fisher’s exact test.

## Results and discussion

**Fig. 2:**
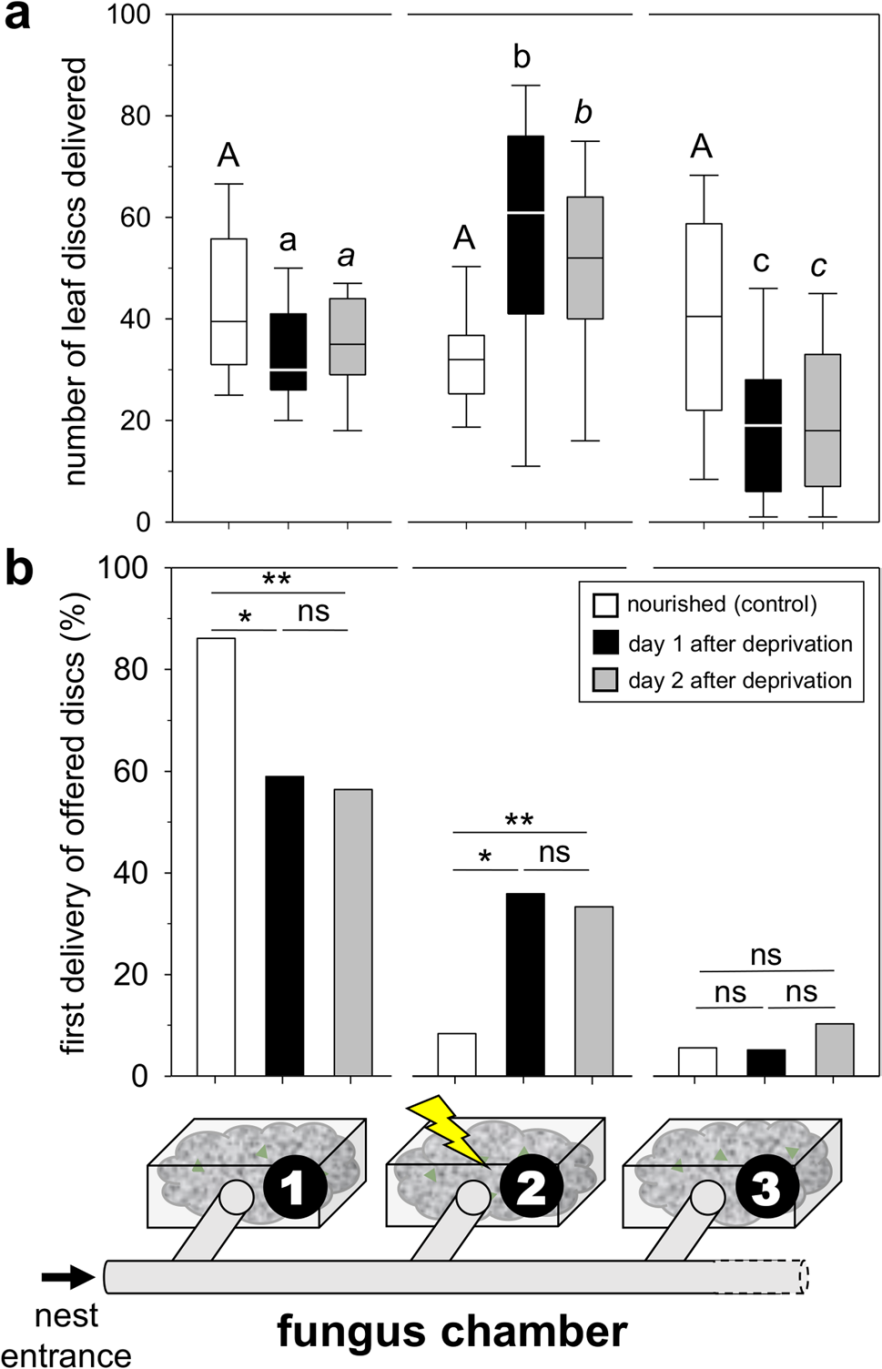
**a** Number of leaf discs delivered to different chambers in both the nourished and undernourished experiments (box: 25–75% percentiles, line: median, whiskers: min–max values; 2-way repeated-measures ANOVA and Kruskal–Wallis test with Tukey post hoc test; boxes sharing the same letters do not differ statistically, *P* ≤ 0.05). Figure legend applies to both **a** and **b**. **b** First delivery of offered discs in each chamber, as percentage of the total delivery of first discs for each condition (*n*_nourished_ = 36, *n*_undernourished day 1_ = 39, *n*_undernourished day 2_ = 39; Fisher’s exact test, **P < 0.01, *P ≤ 0.05, ns = not significant)

Workers readily picked up the offered leaf discs and transported them back into the nest. When all fungus chambers were well nourished, there was no difference in the distribution pattern over the day and all chambers were equally supplied (Fig. 2a, for the sake of uniformity, data are presented as box plots, as data of the ‘nourished’ experiment met normality and equal variance, while the pooled data of the ‘undernourished’ experiment did not; ‘nourished’ experiment: 2-way repeated-measures ANOVA, factor time of day *P* = 0.275, factor chamber *P* = 0.376).

An even resource distribution was no longer the case when one of the fungus gardens experienced undernourishment. While time of day had also no effect on the distribution pattern as in the previous experiment, chambers were supplied differently (Fig. 2a, 2-way repeated-measures ANOVA, factor time of day *P* = 0.61, factor chamber *P* < 0.001). The undernourished chamber 2 received almost twice the number of discs compared to chamber 1 (median_chamber 1_ = 30, median_chamber 2_ = 61), while chamber 3 (median_chamber 3_ = 19) received much less (Fig. 2a, pooled data, time of day 10:00, 12:00, and 14:00 h; Kruskal–Wallis test *P* < 0.001; Tukey post hoc test, all chambers pairwise comparisons, *P* < 0.05). It appears likely that the reduced delivery to chamber 3 resulted from the limited number of discs offered in each assay (100 discs), rather than from a workers’ response aimed at avoiding such chamber, since a higher delivery to one chamber would logically leave less discs to be delivered to another chamber.

Supply of chamber 2 did not lessen 1 day later, and a pattern similar to day 1 could be observed (Fig. 2a, 2-way repeated-measures ANOVA, factor time of day *P* = 0.62, factor chamber *P* < 0.01; pooled data, time of day 10:00, 12:00, and 14:00 h; Kruskal–Wallis test *P* < 0.001; Tukey post hoc test, all chambers pairwise comparisons, *P* < 0.05). The similar distribution pattern of day 1 and day 2 after leaf deprivation could either result from an enduring demand of the fungus, as leaf fragments collected the first day and overnight had not mitigated undernourishment, or because workers continued to respond to orientation cues although the fungus may have recovered. Overall, our results indicate that LCA can perceive the undernourished condition of their fungus and specifically react with a higher supply. In the ant *Temnothorax albipennis*, *Solenopsis invicta*, and *Lasius niger*, workers can also perceive starvation in nestmates and brood, and react with increased liquid uptake (Howard and Tschinkel 1980) or by supplying the same number of workers, or even more workers at a faster speed (Sendova-Franks et al. 2010; Mailleux et al. 2011).

In the ‘nourished’ experiment, most workers delivered the very first of the offered leaf discs to chamber 1, the closest to the nest entrance (Fig. 2b). Chambers 2 and 3 rarely received the first delivery (statistics: Table 1a). In the ‘undernourished’ experiment, however, while chamber 1 still received most of the first delivered discs on day 1 as well as on day 2, clearly more workers chose the undernourished chamber 2 for their first delivery. Statistically significant differences resulted from a change of highest first delivery from chamber 1 (nourished experiment) to chamber 2 (undernourished experiment), while chamber 3 only rarely received the first delivery of the offered leaf discs in either experiment (Table 1b).

**Table 1:** Statistics for the first delivery of offered leaf discs, after Fisher’s exact test: **a** Pairwise comparisons of the overall distribution pattern between different experimental conditions. For each chamber, counts of delivered discs were pooled for the three time points, as there were no statistical differences in the delivery among them; **b** Pairwise comparisons between different experimental conditions, for each chamber

a		Comparison	*P* value	
		Nourished *vs.* undernourished day 1	0.0086	**
		Nourished *vs.* undernourished day 2	0.0107	*
		Undernourished day 1 *vs.* undernourished day 2	0.78	NS
b	Chamber	Comparison	*P* value	
	1	Nourished *vs.* undernourished day 1	0.011	*
	1	Nourished *vs.* undernourished day 2	0.006	**
	1	Undernourished day 1 *vs.* undernourished day 2	1	NS
	2	Nourished *vs.* undernourished day 1	0.011	*
	2	Nourished *vs.* undernourished day 2	0.006	**
	2	Undernourished day 1 *vs.* undernourished day 2	1	NS
	3	nourished *vs.* undernourished day 1	1	NS
	3	nourished *vs.* undernourished day 2	0.68	NS
	3	undernourished day 1 *vs.* undernourished day 2	0.67	NS

Do ants recognize the nutritional state of a fungus garden without entering the chamber? If so, ants should perceive and respond to putative volatiles emanating from the fungus garden via the side tunnel. An undernourished fungus could emit a volatile blend that differs from the emission of a symbiont in a good nutritional condition. Alternatively, leaf supply to chambers could be guided by pheromone markings. Laden LCA mark their foraging trails on their way back to the nest (Robinson et al. 1974). They could continue these markings beyond the nest entrance until they reach a chamber. Other laden workers could follow that trail into the chamber while also laying a pheromone trail, reinforcing the supply by positive feedback until demand in this chamber is satisfied. As the undernourished chamber had a higher demand, trail laying could have lasted longer, attracting more foragers and thus increasing the traffic flow towards that chamber. Future analysis of ant traffic flows under different nourishment levels could help to uncover the involvement of chemical trails in the organization of intra-nest resource distribution.

Interestingly, more foragers bypassed chamber 1 and delivered the first of the offered leaf discs to chamber 2 when that fungus garden was undernourished. This hints at the existence of informed foragers stemming from chamber 2 that supplied leaf discs directly to the fungus garden they originated from. In addition, the increased overall delivery to the undernourished garden could also be caused by the involvement of more workers originating from that chamber, likely with lower foraging thresholds, going out and returning to it loaded with a disc. Importantly, these foragers were not undernourished themselves, as they received diluted honey *ad libitum* during the undernourishment of their garden, so that they appear to respond directly to the state of the fungus. In fact, *Acromyrmex lundii* LCA are known to respond to so far unknown cues from the fungus garden, and to discontinue foraging of leaf materials previously experienced as harmful to the fungus but not to themselves (Herz et al. 2008).

LCA nests may house up to eight thousand fungus chambers (Moreira et al. 2004) that need to be supplied with leaf fragments. In large nests, there can be quite some distance between chambers situated at different locations in the network. Despite this, LCA are able to evenly distribute resources within the nest (Pretto and Forti 2000), so that any distance effect on resource distribution, as observed in the first delivery of offered fragments in our experiment, was transitory and no longer noticeable over time. We propose that overprovisioning of the chambers closest to the nest entrance is avoided via the following mechanisms, which ultimately may result in an even resource distribution: (i) a high delivery to a first chamber may generate congestion of loaded workers at the chamber entrance, and prompt some of them to continue walking downstream to the next chamber, and so a; (ii) loaded foragers may, after having entered a well-provisioned chamber, leave it without unloading and walk downstream; (iii) inside-nest workers may remove and relocate some fragments to the next downstream chambers. In *Atta colombica* LCA, it was observed that fungus gardens located farther from the nest entrance along a series of three chambers were supplied with large delays (Burd and Howard 2005), as expected because of the distance effect. However, the three nest chambers were serially yet directly connected one after another, so that leaf fragments transported downstream had to be carried through the previous chambers, a situation that is not observed in natural nests. Consequently, not only the distance but also the size of the carried fragments was reported to affect the speed of their transport, which is likely an experimental artifact of the unnatural nest arrangement used. Therefore, the hypothesis that the constraints imposed by the underground transport and processing of leaf fragments may have shaped the evolution of fragment size determination in LCA, as advanced by the authors, remains elusive.

As avenues for future research, we hypothesize that LCA achieve a suitable leaf supply to their fungus chambers via the following behavioral mechanisms: (1) informed workers guide supply to chambers with higher resource demand by laying pheromone trails; (2) naïve workers orient towards volatiles emanating from fungus chambers and/or follow pheromone trails laid within the nest; (3) overprovisioning is prevented by relocation of excessive leaf fragments to nest chambers with higher demand, or by temporary storage in leaf caches, from where they are delivered to neighboring fungus chambers.

## Data Availability

The dataset of this study is available in the supplementary material.
